# Thromboembolic Complications after Bariatric and Metabolic Surgery: A Single-Center Experience, Two Case Reports and a Literature Review

**DOI:** 10.15388/Amed.2024.31.2.23

**Published:** 2024-12-04

**Authors:** Žygimantas Juodeikis, Gintautas Brimas

**Affiliations:** 1 Clinic of Gastroenterology, Nephro-Urology and Surgery, Vilnius University, Vilnius, Lithuania Department of Surgery, Republican Vilnius University Hospital, Vilnius, Lithuania

**Keywords:** bariatric and metabolic surgery, sleeve gastrectomy, thromboembolic complications, Raktažodžiai: bariatrinė ir metabolinė chirurgija, skrandžio rezekcija, tromboembolinės komplikacijos

## Abstract

**Background:**

Bariatric and metabolic surgery has emerged as an effective intervention for morbid obesity, offering substantial and sustained weight loss along with improvements in various comorbidities. Among the diverse spectrum of potential postoperative complications, thromboembolic events have garnered increasing attention due to their significant morbidity and mortality.

The aim of this study is to present a single-center experience of thromboembolic complications following bariatric and metabolic surgery. Additionally, we present two distinctive cases, highlighting the clinical manifestations, diagnostic challenges, and therapeutic interventions associated with postoperative thromboembolism.

**Materials and Methods:**

We retrospectively reviewed data from patients with obesity who underwent various bariatric and metabolic operations at Republican Vilnius University Hospital from January 2018 to February 2024. All patients, regardless of the type of operation performed, were included. Two patients with thromboembolic complications are presented as illustrative cases.

**Results:**

A total of 633 patients were included in the analysis: 278 underwent laparoscopic adjustable gastric banding, 345 underwent sleeve gastrectomies, and 10 underwent gastric bypasses. Thromboembolic complications occurred in only two patients, with one developing portal vein thrombosis and the other developing pulmonary embolism.

**Conclusions:**

This single-center experience emphasizes the unpredictable nature of thromboembolic events in postbariatric surgery patients and highlights the critical role of vigilant monitoring, early detection, and individualized therapeutic interventions. Continued research efforts are warranted to refine risk stratification, enhance preventive measures, and improve overall patient outcomes in the landscape of bariatric surgery.

## Abstract (it)

**Įvadas:**

Bariatrinė ir metabolinė chirurgija yra veiksmingiausia intervencija gydant morbidinį nutukimą, reikšmingai ir ilgam laikui sumažinanti kūno masę bei pagerinanti su nutukimu susijusių gretutinių ligų kontrolę. Tromboembolinės komplikacijos pooperaciniu laikotarpiu yra vienos pavojingiausių ir nulemia didžiausią šio ligonių kontingento mirtingumą.

Šio tyrimo tikslas yra pateikti vieno bariatrinės ir metabolinės chirurgijos centro tromboembolinių komplikacijų patirtį. Taip pat pateikiame dvi tromboembolijų klinikines apraiškas.

**Metodai:**

Atlikome retrospektyvią pacientų, kuriems Respublikinėje Vilniaus universitetinėje ligoninėje nuo 2018 m. sausio iki 2024 m. vasario buvo atliktos įvairios bariatrinės ir metabolinės operacijos, duomenų analizę. įtraukti visi pacientai nepriklausomai nuo atliktos operacijos tipo. Du pacientai, kuriems buvo tromboembolinės komplikacijos, yra pristatyti kaip klinikiniai atvejai.

**Rezultatai:**

į analizę įtraukti 633 pacientai: 278 pacientams atliktas laparoskopinis skrandžio apjuosimas reguliuojama juosta, 345 pacientams atlikta vertikali skrandžio rezekcija ir 10 pacientų – skrandžio ir tuščiosios žarnos apylankos operacija. Tromboembolinės komplikacijos nustatytos dviem pacientėms: vienai pacientei išsivystė vartų ir blužnies venų trombozė, kitai – plaučių arterijos embolija.

**Išvados:**

Šis tyrimas pabrėžia tromboembolinių komplikacijų nenuspėjamumą bei budraus stebėjimo, ankstyvos diagnostikos ir individualizuoto gydymo svarbą bariatrinėje ir metabolinėje chirurgijoje. Siekiant sumažinti tromboembolinių komplikacijų riziką tikslinga tobulinti rizikos vertinimo metodikas bei standartizuoti prevencines priemones.

## Introduction

Bariatric surgery has emerged as a transformative intervention for individuals with morbid obesity, offering substantial and sustained weight loss alongside amelioration of obesity-related comorbidities. The growing prevalence of bariatric procedures worldwide attests to the increasing recognition of their efficacy in enhancing both quality of life and overall health. However, the benefits of bariatric and metabolic surgery are accompanied by inherent risks, one of the notable concerns being the development of thromboembolic complications.

Venous thromboembolism (VTE), comprising deep vein thrombosis (DVT) and pulmonary embolism (PE), poses a significant postoperative challenge in bariatric surgery patients. The complex interplay of obesity-related physiological alterations, surgical trauma, and the inherent prothrombotic state of obesity collectively contribute to an elevated risk of thromboembolic events in this patient population. Despite advancements in surgical techniques and perioperative care, the incidence and implications of thromboembolic complications persist, warranting a meticulous examination of their prevalence, risk factors, and management strategies.

The aim of this study is to present a single-center experience of thromboembolic complications following bariatric and metabolic surgery, synthesizing current knowledge on prevalence, risk factors, and management strategies. Additionally, we present two distinctive cases, highlighting the clinical manifestations, diagnostic challenges, and therapeutic interventions associated with postoperative thromboembolism.

## Materials and methods

We conducted a retrospective review of medical records for patients who underwent various bariatric procedures in Republican Vilnius University Hospital between January 2018 and February 2024. Patient demographics, as well as the date and type of procedure performed, were collected. Additionally, we examined all medical data up to one month after the operation to determine if patients developed thromboembolic complications after discharge from the hospital.

Statistical analysis was performed using SPSS version 21.0 (SPSS Inc., Chicago). Values were expressed as means with standard deviation.

## Results

A total of 633 patients were included in the analysis, among whom 278 underwent laparoscopic adjustable gastric banding, 345 underwent sleeve gastrectomies, and 10 underwent gastric bypasses. The operations were performed by three bariatric and metabolic surgeons who had completed their learning curves some time ago. The average operating times were 65 minutes for gastric banding, 75 minutes for sleeve gastrectomy, and 140 minutes for gastric bypass. The baseline characteristics of the patients are depicted in Table 1. Thromboembolic complications occurred in two (0.31%) patients, with one developing portal vein thrombosis and the other pulmonary embolism.

**Table 1:** Baseline characteristics

Number of patients	633
Age (years)	44.8 ±11.5
Gender:	
Male (%)	199 (31.4)
Female (%)	434 (68.5)
Body weight (kg)	132.3 ±21.7
BMI (kg/m^2^)	48.1 ±8.2

Values are expressed as standard deviation.

## Case Presentations

### Case 1: 44-Year-Old Woman (Operated on January 11, 2024)

The first patient was a 44-year-old woman with a BMI of 44 and a history of smoking. She had no prior abdominal surgeries and no comorbidities. On January 11, 2024, she underwent a laparoscopic sleeve gastrectomy, which was completed without any intraoperative complications. The total operative time was 1 hour and 20 minutes.

As part of standard perioperative management, the patient received 3500 IU of bemiparin subcutaneously preoperatively and again at 8 pm. Upon returning to the surgical ward, she reported no severe pain, was allowed to drink fluids, and was encouraged to mobilize early.

The following day, at 5 am, the patient became cyanotic and experienced cardiac arrest. Resuscitation efforts were initiated immediately, and the patient was transferred to the intensive care unit (ICU). Despite all measures, resuscitation was unsuccessful, and biological death was declared one hour later.

An autopsy performed the next day revealed a massive pulmonary artery embolism.

### Case 2: 39-Year-Old Woman (Operated on September 25, 2023)

The second case involves a 39-year-old female patient with a BMI of 35. Similar to the first case, she had no prior abdominal surgeries but presented with well-controlled arterial hypertension. On September 25, 2023, the patient underwent a sleeve gastrectomy without encountering any intraoperative complications. The total operative duration was 1 hour and 30 minutes.

As part of the standard perioperative protocol, the patient received 3500 IU of bemiparin subcutaneously preoperatively and again at 8 pm. After returning to the surgical ward, she had no complaints and was encouraged to mobilize early.

**Figure 1:**
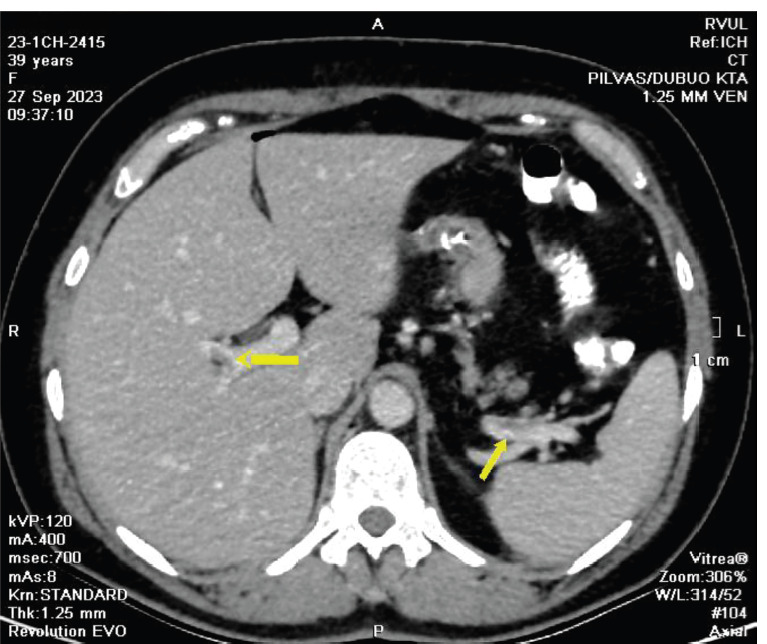
CT scan demonstrates portal and splenic vein thrombosis.

On the first postoperative day, fluoroscopy revealed no signs of leaks. With a CRP level of 48, the patient was permitted to resume oral intake. However, on the second day following the operation, a notable elevation in CRP levels was observed, measuring 282. A double-contrast CT scan was conducted, unveiling evidence of thrombosis in the portal vein and splenic vein (Figure 1). Consequently, therapeutic measures included the administration of low molecular weight heparins and analgesics. The patient was discharged from the hospital on September 30, with CRP levels measuring 124. Additionally, she was prescribed oral anticoagulants in the form of edoxaban 30 mg, for a duration of six months. During a controlled visit after 3 months the patient had no complaints, with normal blood test results.

## Discussion

Numerous studies identified obesity as an independent risk factor for VTE [1]. VTE complications, while relatively rare, remain a leading cause of mortality after bariatric and metabolic surgery [2,3]. The diverse manifestations of thromboembolic complications underscore the multifaceted nature of postoperative challenges in this patient population. Our presented cases highlight the need for a comprehensive understanding of the risk factors associated with specific surgical techniques and the importance of tailoring preventive measures accordingly. Furthermore, heightened awareness, early detection, and individualized management strategies tailored to the specific clinical scenario are paramount.

Based on data from multiple databases, the overall incidence of VTE, including deep vein thrombosis (DVT) and PE, ranges from 0.17% to 0.4% [4,5]. Additionally, most VTEs occur after discharge; 2% occur intraoperatively, and 25% occur before hospital discharge.

Based on the experience reported by a large multi-institutional referral center, the incidence of venous thromboembolism varies between different bariatric procedures [6]. The VTE rates were 1.1% for gastric bypass, 2.9% for sleeve gastrectomy, and 0.2% for gastric banding. The highest rates of VTE complications are reported in patients undergoing biliopancreatic diversion and revisional procedures.

In our study, the rate of thromboembolic events was 0.31%, which is consistent with reported incidences.

Risk factors for VTEs include a body mass index (BMI) >50 kg/m^2^, a history of VTE, a history of hypercoagulable disorders, pulmonary hypertension, venous stasis disease, poor functional status, open or revision surgery, and operative time >3 hours [4]. The most common risk factors associated with fatal PE include severe venous stasis disease, BMI >60 kg/m^2^, truncal obesity, and obesity-hypoventilation syndrome. Patients undergoing revision bariatric surgery are at a higher risk of VTE.

Preoperative risk stratification can be a useful tool for identifying high-risk patients who may benefit from more aggressive prophylaxis. For patients identified preoperatively as high risk for PE, due to previous medical history of VTE, PE, venous stasis disease, or inability to ambulate, perioperative chemoprophylaxis with low molecular weight heparin or unfractionated heparin may be prescribed for an extended period, even after hospital discharge. Given that the average time to developing VTE is 21 to 28 days, many bariatric surgeons continue chemoprophylaxis for six weeks postoperatively.

There is no consensus on the optimal preventive measures for thromboembolic complications in bariatric patients. Various guidelines recommend measures such as subcutaneous unfractionated or low molecular weight heparin (LMWH), intermittent compression devices, compression stockings, and early mobilization. The current American Society for Metabolic and Bariatric Surgery guidelines for thromboprophylaxis recommend that all bariatric patients receive mechanical prophylaxis and are encouraged to ambulate early [7].

As a result, most bariatric centers use their own algorithms for preventing thromboembolic complications. At our hospital, the standard protocol includes administering LMWH twice daily and encouraging early mobilization. Additionally, LMWH is prescribed for ten days following discharge.

Evidence suggests that prolonged operative times are associated with adverse surgical outcomes across various procedures [8,9]. Specifically, extended operative durations have been linked to an increased incidence of VTE events following laparoscopic gastric bypass [10].

In the cases we presented, the operation times were 80 and 90 minutes, which are 5 and 15 minutes longer than the average sleeve gastrectomy times at our hospital. These prolonged operation times may indicate that the surgeons encountered some difficulties during the procedures.

There is growing evidence that inflammatory markers may serve as potential predictors of complications in postbariatric and metabolic surgery patients [11,12]. At our hospital, we routinely measure white blood cell counts and CRP levels on the first and second postoperative days. Serum CRP levels above 200 mg/L prompt further investigation of the patient, depending on the individual situation.

Retrospectively evaluating the very rapid onset of symptoms – sudden cyanosis and cardiac arrest – presented in Case 1, and considering the autopsy results indicating massive PE, it is evident that there was no chance to save the patient. The unexpected development of PE in a patient without any obvious risk factors, except for smoking, underscores the need for further research into the predisposing factors and preventive strategies for such complications in bariatric and metabolic surgery.

The presentation of thrombosis in the vena portae and splenic vein in a second case following a seemingly uneventful sleeve gastrectomy highlights the multifaceted nature of thromboembolic complications in bariatric surgery. The elevated CRP levels on the second day postoperatively triggered prompt diagnostic imaging, allowing for an early diagnosis and initiation of therapeutic anticoagulation. The discharge of the patient with reduced CRP levels suggests a positive response to anticoagulation therapy, reinforcing the significance of timely management in mitigating thromboembolic progression.

The uneventful nature of the surgical procedures in both cases underscore the challenges in predicting and preventing thromboembolic complications based solely on preoperative, intraoperative and immediate postoperative assessments. Comprehensive postoperative monitoring, particularly for patients with identifiable risk factors, is paramount to ensure early detection and intervention. These cases contribute to the growing body of literature on thromboembolic complications in bariatric and metabolic surgery, prompting further exploration into risk factors, preventative measures, and optimal management approaches to enhance patient safety in this evolving field.

It is crucial to acknowledge the limitations inherent in individual case reports and the retrospective nature of this study. There is still a possibility that some patients developed asymptomatic or mildly symptomatic VTE complications after discharge, which were not diagnosed and treated properly.

Further investigations are required to determine the true incidence of these complications, identify specific risk factors, and establish optimal management strategies for thromboembolic complications in the diverse landscape of bariatric and metabolic surgery.

## Conclusions

In conclusion, the presented cases emphasize the unpredictable nature of thromboembolic events in postbariatric surgery patients and highlight the critical role of vigilant monitoring, early detection, and individualized therapeutic interventions. Continued research efforts are warranted to refine risk stratification, enhance preventive measures, and improve overall patient outcomes in the dynamic landscape of bariatric and metabolic surgery.
